# Gene regulatory network and abundant genetic variation play critical roles in heading stage of polyploidy wheat

**DOI:** 10.1186/s12870-018-1591-z

**Published:** 2019-01-03

**Authors:** Chaonan Shi, Lei Zhao, Xiangfen Zhang, Guoguo Lv, Yubo Pan, Feng Chen

**Affiliations:** National Key Laboratory of Wheat and Maize Crop Science/Agronomy College, Henan Agricultural University, 15 Longzihu College District, Zhengzhou, 450046 China

**Keywords:** Polyploidy wheat, Heading stage, Vernalization, Photoperiod, Gene regulatory network, Genetic variation

## Abstract

**Background:**

The extensive adaptability of polyploidy wheat is attributed to its complex genome, and accurately controlling heading stage is a prime target in wheat breeding process. Wheat heading stage is an essential growth and development processes since it starts at a crucial point in the transition from vegetative phase to reproductive phase.

**Main body:**

Heading stage is mainly decided by vernalization, photoperiod, hormone (like gibberellic acid, GA), and earliness per se (*Eps*). As a polyploidy species, common wheat possesses the abundant genetic variation, such as allelic variation, copy number variation etc., which have a strong effect on regulation of wheat growth and development. Therefore, understanding genetic manipulation of heading stage is pivotal for controlling the heading stage in wheat. In this review, we summarized the recent advances in the genetic regulatory mechanisms and abundant variation in genetic diversity controlling heading stage in wheat, as well as the interaction mechanism of different signals and the contribution of different genetic variation. We first summarized the genes involved in vernalization, photoperoid and other signals cross-talk with each other to control wheat heading stage, then the abundant genetic variation related to signal components associated with wheat heading stage was also elaborated in detail.

**Conclusion:**

Our knowledge of the regulatory network of wheat heading can be used to adjust the duration of the growth phase for the purpose of acclimatizing to different geographical environments.

**Electronic supplementary material:**

The online version of this article (10.1186/s12870-018-1591-z) contains supplementary material, which is available to authorized users.

## Background

Wheat (*Triticum aestivum* L*.*) is widely cultivated across the globe, where it has adapted to different environments as a result of its natural diversity and complex genome. Bread wheat is a polyploid species with an AABBDD genome, and genome A, B, and D respectively originated from *T. urartu*, *Aegilops speltoides,* and *Ae. Tauschii* [1–3]. Various cultivars of wheat possess different growth characteristics to endure external stress and adapt to different climatic conditions and geographical environments through regulating their heading stage [4].

Heading occurs in the transition of wheat from the vegetative stage to the reproductive stage. As one of the most important agronomic traits of wheat, the heading days of each cultivar were calculated from the sowing date to the date of more than half of the plants has been heading [5]. The duration of the heading stage determines the flowering time of wheat, which subsequently impacts wheat yield. Vernalization, photoperiod, and exogenous hormones constitute exogenous factors that influence heading stage, while endogenous hormone and narrow-sense earliness per se (*Eps*) as an endogenous factor influences the duration of the wheat heading stage [6–12].

## Main text

### Part 1. Different signals cross-talk to control wheat heading stage

Interaction among the genes related to vernalization, photoperiod, hormone, and *Eps* to construct a regulatory network determine wheat growth and development as well as the duration time of the heading stage [7, 11–13]. The molecular regulatory network of flowering is well understood in the diploid plant *Arabidopsis*, but remains poorly understood in polyploidy wheat, especially bread wheat which is a long-day hexaploid species. Over the past decades, an increasing number of molecular genetic studies have extended our understanding of the wheat flowering regulatory network. Here, we summarize previous works to further ascertain the genetic networks and epigenetic chromatin modifications in the control of wheat heading stage. The molecular mechanisms of signal cross-talk and allelic variation of related genes in heading stage control in bread wheat are complex, and we hope to provide a relatively complete view of the complex network of wheat heading stage regulatory pathways.

### Vernalization signaling regulatory network in wheat

**Fig. 1:**
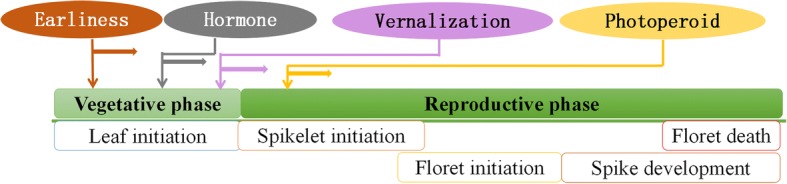
Wheat heading stage are affected by multi-environment. Based on the growth habit of winter wheat, hormone and earliness per se *(Eps)* can work on the whole life of the wheat growth and development, vernalization always being to take effect on the transition from the vegetative phase to reproductive phase during winter time, following by vernalization, photoperiod functioned to control flowering. Different colors represent different signals

Certain types of plants go through a period of sustained low temperature to promote flowering, namely vernalization [14]. Wheat is a typical crop that requires vernalization to accelerate flowering. Based on growth habit, wheat cultivars with a spring growth habit have no vernalization requirement and can be planted in spring, are classified as spring wheat. Wheat cultivars that planted in autumn and can flower during the subsequent spring are regarded as winter wheat and require vernalization to ensure punctual flowering [15]. In regard to winter wheat with predominant planting area, it suggests that different signals start at different developmental stages based on its growth habit. For example, endogenous hormone and *Eps* play important roles throughout the growth and development process, and then vernalization, photoperoid and exogenous hormone have a good chance to work in later period of wheat development, as indicated in Fig. 1. The most obvious difference of them is their heading stage response when deal with vernalization or developing in a long-day condition. Vernalization can facilitate the transition from the vegetative stage to the reproductive stage and minimize exposure to harsh surroundings, thereby maintaining a stable output.

The wheat vernalization regulatory network is mainly controlled by three pivotal genetic loci: *Vernalization1* (*Vrn-A1, Vrn-B1, Vrn-D1*)*, Vernalization2* (*Vrn-A2*)*,* and *Vernalization3* (*Vrn-B3*) [16–18]*.* These genes have been cloned and validated to directly or indirectly take part in the vernalization pathway. *Vrn1* was cloned by chromosome walking in diploid wheat and encodes a MADS-box transcription factor, which is homologous to the *Arabidopsis APETALA1* with a protein-binding site (CArG-box) in the promoter region [16]. *Vrn1* can directly activate *Vrn-B3* expression under LD conditions [19, 20]. It could be significantly up-regulated by prolonged exposure to a cold environment and directly accelerates the transition to reproductive development at the shoot apex, but *Vrn1* is not essential gene of wheat flowering since *Vrn1* mutants (with premature stop codon of *Vrn-A1*, *Vrn-B1* and *Vrn-D1*) can flower and produce seeds under both vernalized and unvernalized conditions, suggesting the existence of other wheat flowering association genes that make wheat cultivars preferable adapted to vagaries of climate [21]. When a winter wheat carries the 3 recessive *vrn-1* (*Vrn-A1*, *Vrn-B1* and *Vrn-D1*) alleles, transition is significantly accelerated by vernalization treatment [22, 23]. Voss-Fels et al. (2018) showed that six SNP markers of a highly significant QTL for nodal root angle index on chromosome 5B was identified to have strong linkage disequilibrium to *Vrn-B1*, and thus speculate that *Vrn1* was possibly involved in the regulation of plant morphology in wheat and barley [24]. In addition, *Wheat vegetative to reproductive transition-1* (*TaVRT-1*) and *Wheat APETALA1* (*WAP1*) were identified to be highly similar to *Vrn1,* and these three genes maybe the same locus. They were identified on chromosome 5A, 5B and 5D, and encode an *APETALA1* (*AP1*)*-*like MADS box gene. They are all not only induced by vernalization and photoperiod but also are involved in the phase transition and are maintained during the reproductive phase [16, 22, 23].

Histone modification is an important post-translational process in epigenetic regulation, and it can influence various phenotype by changing gene expression [25, 26]. Methylation modification of histone lysine mainly occurs in the 4, 9, 27, and 36 lysine residues of histone H3 in eukaryotes. H3K9me and H3K27me have been confirmed to be related to the inhibition of gene expression, but H3K4me and H3K36me were related to the activation of gene expression [27]. Vernalization activated the *Vrn1* transcript by changing the ratio of H3K4me3 and H3K27me3. Vernalization enhanced the H3K4me3 expression level of *Vrn1* and *Vrn3* with no concurrent change in H3K27me3 in winter wheat. *Vrn2* repression is associated with methylation and a lower H3K27me3 methylation level, but the enrichment of H3K27me3 may have a role in repressing *Vrn2* [28]. In barley, however, *Vrn1* transcripts were up-regulated during vernalization regardless of whether under long or short days, whereas the expression levels of *Vrn3* and *Vrn2* had no obvious changes under short days. It is thus speculated that *Vrn1* is a direct target gene of vernalization. Researchers found that vernalization started the epigenetic regulation of the *Vrn1* chromatin state, but no similar phenomenon was observed in *Vrn2* or *Vrn3*, further verifying that *Vrn1* is a direct target gene of vernalization. In *Arabidopsis*, vernalization-induced flowering is mediated by the epigenetic regulation of the floral repressor *FLC*, but in barley or other cereals, is mediated by the epigenetic regulation of the floral activator *Vrn1*. Vernalization alters the methylation level of *Vrn1* and increases the levels of the active histone 3 lysine 4 trimethylation (H3K4me3) and suppresses the levels of histone 3 lysine 27 trimethylation (H3K27me3) [29].

*Vrn2*, initially mapped on chromosome 5A, as a dominant repressor of flowering time that is downregulated by vernalization, is composed of two tandem duplicated CCT domain (*CONSTANS*, *CO-like*, and *TOC1*) genes *ZCCT1* and *ZCCT2* with a putative zinc finger in the N-terminus and a CCT domain in the C-terminus, and reduction of the expression of *ZCCT1* is closely associated with the acceleration of flowering time. They have no orthologs of *Vrn2* in rice or *Arabidopsis*, so *Vrn2* may be a special regulatory element during the evolution of wheat [17]. *Vrn3* encodes a RAF kinase inhibitor–like protein that positively regulates wheat flowering and shows high similarity to *FLOWERING LOCUS T* (*FT*) in *Arabidopsis*. *Vrn3* is induced by long days and acts as a bridge linked to the vernalization and photoperiod pathway, which then further accelerates reproductive apex development [18]. Therefore, an active *Vrn1* or *Vrn3* can accelerate flowering, but the activation of *Vrn2* requires vernalization in plants to flower. *Vrn-D4,* derived from the insertion of part of the long arm of chromosome 5A (including *Vrn-A1*) into the short arm of chromosome 5D, is a homologous gene of *Vrn1*. As another vernalization-related gene, *Vrn-D4* encodes a protein with high similarity to the *Arabidopsis* meristem identity protein *APETALA1* (*AP1*). This gene was first identified in an Australian wheat cultivar Gabo that made an important contribution to the development of the spring growth habit in ancient wheat from South Asia [30, 31]. Detailed information on the participation of these genes in vernalization pathways was shown in Table 1.

**Table 1:** The detailed information of genes association with the wheat heading-flowering regulatory network

Gene	Chromosomr. location	Protein product	Description	Functions	Mutant phenotype	Gene number in IWGSC1.0	Position in IWGSC1.0	References
*Vrn1*	5A, 5B, 5D	Encodes a MADS-box transcription factor, also named as *WAP1* or *TaVRT1*	Induced by prolonged cold, long photoperiod	Not only promotes the apex transition to generative development, but also activation of long day response in leaves	Recessive alleles at all *Vrn-1* homoeoloci confer a winter growth habit (vernalization sensitive), whereas one or more dominant alleles at *Vrn-1* homoeoloci result in a spring growth habit (vernalization insensitive).	TraesCS5A01G391700, TraesCS5B01G396600, TraesCS5D01G401500.	5A:587423056–587,423,240, 5B:573815719–573,815,903, 5D:467184094–467,184,278.	[16, 22, 23]
*Vrn2*	5A	Encodes a protein containing a putative zinc finger and a CCT protein-protein interaction domain	Down-regulated when plants are vernalized	A dominant repressor of flowering	Functional mutations in the *ZCCT* genes result in a spring growth habit and early flowering	No	No	[17]
*Vrn3*	7B	Encodes a mobile protein, homologous to the *Arabidopsis FLOWERING LOCUS T (FT)*	Induced by vernalization and long days	Accelerates reproductive apex development	Transgenic wheat plants overexpressing *Vrn-3B* have an extra-early flowering phenotype without the need of vernalization	TraesCS7B01G013100	7B:9700818–9,704,363	[18]
*Vrn4*	5D	Encodes a MADS-box transcription factor highly similar to VRN1	*VRN-D4* locus originated by the insertion of a large segment from chromosome arm 5AL into chromosome arm 5DS	*Vrn-D4* likely operates upstream of the positive regulatory feedback loop connecting *Vrn1, Vrn2* and *Vrn3*.	The mutation flowered later than plants carrying the wild type allele	No	No	[30, 31]
*VER2*	2D	Encodes a nucleocytoplasmic carbohydrate-binding protein, a jacalin-like lectin, with high affinity for glcnac and galactose	After vernalization, VER2 accumulates predominantly in the nucleus in shoot tips and young leaves	Nuclear-localized VER2 interacts with O-glcnac-modified TaGRP2 to relieve the repression on tavrn1 transcript accumulation and to promote flowering in hexaploid winter wheat	*VER2* downregulated in winter wheat results in flowered later	No	No	[47, 50]
*TaGRP2*	No.	Glycine-rich RNA binding protein	TaGRP2 is dynamically O-glcnacylated during vernalization	TaGRP2 binds to the pre-mRNA of *Vrn1* and inhibits *Vrn1* expression	*TaGRP2-Ri* plants accelerated flowering compared with wild type	No	No	[50, 51]
*TaVRT2*	7A, 7B, 7D	Encodes a predicted protein of 226 amino acids, belongs to the StMADS 11	Accumulate in winter wheat during the vegetative phase and decline towards the transition to the reproductive phase.	The presence of *Vrn2* and *TaVRT-2* transcripts early during long time exposure could reduce or delay the expression of *Vrn1*	Mutation of *TaVRT-2* results in the advance of flowering	No	No	[48]
*TaGI*	3A, 3B, 3D	Encodes a nucleoplasmically localized protein which contains 1174 amino acid residues	The patterns of *TaGI* rhythmic expression in leaves are regulated by circadian clocks, but can be disturbed by light/dark cycle	Functions in mediating photoperiodic flowering, controlling circadian rhythms and phytochrome signaling	Mutations in the *TaGI* gene cause delayed flowering only in long day photoperiod	TraesCS3A01G116300; TraesCS3B01G135400; TraesCS3D01G118200.	3A:84189859–84,191,364; 3B:117928502–117,930,007; 3D:71969619–71,970,784.	[63, 64]
*WSOC1*	4DL	Is a member of the monocot *SOC1-like* gene family	*WSOC1* expression is affected neither by vernalization nor photoperiod, whereas it is induced by gibberellin at the seedling stage	WSOC1 functions as a flowering activator like SOC1 in *Arabidopsis*	Downregulated of *WSOC1* results in delayed flowering	TraesCS4D01G341700	4D:498394464–498,398,154	[71]
*WPCL1*	No.	Encodes a MYB transcription factor belonging to the GARP family	Maybe regulated by circadian clock	Controlling the early flowering phenotype in the einkorn wheat mutant	Deletion of *WPCL1* leads to flowering even under short-day conditions	No	No	[60, 61]
*TaHD1*	6A, 6B, 6D	Encodes a transcription factor with zinc finger motif and nuclear localization signals, also called *CO2*	Can regulated by long-day condition and circadian clock	Directly regulate vernalization gene under long-day condition	The *co* mutant show a delayed flowering response under long-day environment	TraesCS6A01G289400; TraesCS6B01G319500; TraesCS6D01G269500.	6A:521453035–521,453,945; 6B:567398838–567,399,457; 6D:379572052–379,572,962.	[44, 62]
*TaPHYC*	5A, 5B, 5D	No	Long-day induced wheat PHYC forms signaling active homodimers and translocate into the nucleus	Promotes wheat flowering under inductive photoperiods	The loss of function of wheat *PHYC* results in altered expression of circadian clock and photoperiod genes and a dramatic delay in flowering under long days	TraesCS5A01G391300; TraesCS5D01G401000; TraesCS5B01G396200.	5A:586595153–586,599,481; 5D:466221190–466,223,373; 5B:573216947–573,219,055	[60, 61]
*Ppd1*	2A, 2B, 2D	Encodes a pseudo-response regulator (PRR) protein with a CCT domain, also named *taprr37*	Induced by long-day	Can regulated vernalization genes and participate in circadian clock function	Knockdown of *Ppd1* made the wheat delayed flowering	TraesCS2A01G081900; TraesCS2D01G079600	2A:36936362–36,938,400; 2D:33952488–33,955,629.	[8, 57]

Before vernalization, the expression level of *Vrn1* and *Vrn3* are low; a phenomenon that has a negative effect on wheat flowering. However, prolonged cold exposure increases *Vrn1* transcripts to promote rapid flowering both under short and longer days, and the higher expression of *Vrn1* at the shoot apex can facilitate the development and differentiation of wheat inflorescence meristems, whereas the expression of *Vrn1* in the leaves participates in the long-day flowering response pathway. However, the expression level of *Vrn2* decreases after vernalization. At the low *Vrn2* expression level, long days induce the expression of *Vrn3/TaFT1*. *Vrn1*, *Vrn2*, *Vrn3,* and some other association genes are involved in a positive regulatory feedback loop that aims to balance the expression levels of positive and negative regulatory elements, finally regulating the transition of the apex from the vegetative stage to the reproductive stage [32–36]. *Vrn1* can bind the promoter of *Vrn3* in vernalized plants, and also can target *Vrn2* that were repressors of flowering that are down-regulated in vernalized plants [33, 37]. As a duplicated copy of *Vrn-D1*, *Vrn-D4* is pivotal for ancient wheat from South Asia to develop a spring growth habit, and expresses in the leaves and accumulates after prolonged exposure to low temperature. Furthermore, *Vrn-D4* is expressed earlier than other *VRN1* genes in the absence of vernalization, and induced mutations in this gene resulted in delayed flowering [31]. *NUCLEAR FACTOR-Y* (*NF-Y*) belongs to the *HEME ACTIVATOR PROTEIN* (*HAP*) transcription factor family. Previous studies showed that there were different NF-Y proteins involved in the integration of wheat vernalization and the photoperiod pathway [39–41]. Prior to vernalization treatment, the products of *Vrn2* competed with other CCT-domain proteins (such as the photoperiod gene *CO2,* which is the functional homolog of *CO* in wheat) to interact with *NF-Y* transcription factors to inhibit the transcription of *Vrn3*. Further influence of this interaction is to facilitate or postpone flowering depending on the subunits in the NF-Y complex and the new formation of the complex including the type of CCT domain proteins. In rice and *Arabidopsis*, the FT protein acts as a long-distance flowering signal (florigen) that moves from the leaves to the shoot apical meristem via the phloem and promotes flowering in a wide range of plant species [42, 43]. *TaFDL*, a homologous gene of *FD/OsFD1*, exists in the stem apical meristem of bread wheat, and the VRN3 protein can form a functional protein complex with TaFDL to bind the CArG box domain which is located in the promoter region of *Vrn1* in vitro, leading to transcriptional activation of *Vrn1* [44].

Another vernalization-induced gene *vernalization-related 2* (*VER2*) was identified to encode a nucleocytoplasmic carbohydrate-binding protein. VER2 constitutes a jacalin-like lectin and has a close relationship with GlcNAc and galactose. This gene not only impacts wheat flowering, but also is involved in the regulation of spikelet development. Prolonged exposure to low temperatures enhances the integral O-GlcNAcylation levels. However, if plants are subjected to high temperatures following cold treatment, the integral O-GlcNAcylation levels in plants will decrease. *VER2* accumulated in the nucleus in the shoot tips and young leaves when wheat long-term exposure to low temperature [45–47], and VER2 can specifically recognize O-GlcNAc-modified proteins in vernalized wheat plants. *Wheat vegetative to reproductive transition-2* (*TaVRT-2*) is a member of the *StMADS-11* clade of flowering repressors and encodes a predicted protein of 226 amino acids that is also involved in the flowering pathway in wheat. In addition to the existing conserved domains in the TaVRT-2 protein is an MIKC structure (M, MADS domain; I, intervening region; K, K box; C, C-terminal domain), as well as another conserved bipartite nuclear targeting sequence in the MADS domain, and several putative phosphorylation sites [48].

*T. aestivum glycine-rich RNA binding protein 2* (*TaGRP2*) encodes an RNA-binding protein and *AtGRP7* is an orthologous gene in *Arabidopsis* involved in the direct binding to transcripts of genes that participate in flowering regulation and resistance to stress conditions [49]. In the first intron of the *Vrn1* pre-mRNA, there is a critical regulatory region that is regarded as the RNA binding site following transcription. Before vernalization, *TaGRP2* can directly bind to this binding site of *Vrn1* to prevent transcript accumulation. After vernalization, the role of the O-GlcNAc signaling-mediated *Vrn1* transcripts in the flowering transition stage in winter wheat can be activated. Phosphorylated VER2 (VER2-P) transfers into the nucleus and then gathers in the shoot tips and young leaves, physically interacting with the RNA-binding protein TaGRP2 that is O-GlcNAc modified, and the nuclear-localized VER2 interacts with the O-GlcNAc-modified TaGRP2 to decrease the inhibitory action on *Vrn1* expression [50, 51]. Furthermore, SNP at the binding site of the TaGRP2 protein in the *Vrn-A1* first intron was significantly associated with heading date after vernalization, and plants with 3 SNPs at this binding site headed significantly earlier than those with 1 SNP [52].

Additionally, prior to vernalization in winter wheat, *TaVRT2* (*VEGETATIVE TO REPRODUCTIVE TRANSITION 2*) encodes a MADS box gene with sequence similarity to the *Arabidopsis* SHORT VEGETATIVE PHASE (SVP) [48], which is regulated independently by photoperiod and vernalization and accumulates during the vegetative phase. It can also directly bind the CArG box of the *Vrn1* promoter in vivo to inhibit its activity, and this inhibition is enhanced by VRN2. Once vernalized, the expression of *TaVRT2* and *Vrn2* are repressed, thus resulting in the gradual accumulation of *Vrn1*. In the shoot apical meristem (SAM), the activation of *Vrn1* induces the transition of the stem apical meristem from the vegetative to the reproductive phase [53].

### Photoperiod and other factors cross talk with vernalization signaling pathways

Based on the ability of reproductive growth to be initiated by photoperiod, crops can be classified into long day (LD) or short day (SD) species. LD plants can promote flowering by an over-threshold day length, but a sub-threshold day length can make SD plants flower. Plants growing under long day conditions can flower faster by boosting the transition to reproductive growth. As a long-day crop, wheat can adapt to a broad range of climatic and environmental conditions due to its insensitivity to photoperiod (day length). Wheat can punctually flower when growing under SD conditions (illumination time less than 10 h) under vernalization, and the plants foretell external environmental changes and adjust the flowering time appropriately through photoperiod information [54]. Plants have its own photoreceptors to perceive light signaling, and they can also accommodate the rhythm of light and dark to readjust their circadian clock. Research on flowering time controlling in *Arabidopsis* also not only concerned day length, but also photoreceptors and circadian clock [55, 56]. Many major components have been identified in *Arabidopsis*, but only a handful of homologous genes have been cloned in wheat, and the molecular mechanism of the photoperiodic network in wheat is not clear.

In wheat, the photoperiodic regulation of flowering is determined by the dominant genes *Photoperoid-A1* (*Ppd-A1*), *Photoperoid-B1* (*Ppd-B1*), and *Photoperoid-D1* (*Ppd-D1*) located on chromosomes 2A, 2B, and 2D, respectively, and they can reduce the retardation of heading stage under SD conditions by controlling wheat sensitivity to photoperiod [54]. The barley *Ppd-H1* gene has been cloned by positional cloning and codes for a pseudo-response regulator (PRR) protein with a CCT domain as a kind of genes were involved in circadian clock. The recessive *Ppd-H1* barley mutation exhibits a later flowering time under long-day, but with no short-day phenotypic effect [8, 57]. *Ppd-D1a* is a homologous gene of the *Ppd-H1* in hexaploid wheat with early flowering, other than barley, the semi-dominant *Ppd-D1a* wheat mutation has a phenotype that flowers rapidly in short-day or long-day environments [57]. In addition, *Ppd-1* has a close relationship to the development of wheat spikelet via promoting the expression level of *Vrn3* that is a hub between these two pathways, and the *Ppd-D1* is the main active site to regulate wheat heading and flowering dates in the Yellow and Huai winter wheat region, followed by *Vrn-B1* and *Vrn-D1* [5, 58]. Early studies discovered that the factors that influence photoperiodic flowering include day length and different photoreceptors. When a red/far-red-light photoreceptor has an optical signal that contains red light, this facilitates the accumulation of physiologically active Pfr (PHYB:PHYC heterodimers and PHYC:PHYC homodimers). Under far-red light, Pfr is transformed into the devitalized Pr form. PHYTOCHROME C (PHYC) can independently activate the transcription of *PPD1* and the circadian clock output genes *CO2/TaDH1* under irradiation [59, 60]. *GZ3260* is detected in einkorn wheat and encodes a member of the MYB transcription factor family and is a homolog of *PCL1* and *LUX* in rice and *Arabidopsis*, respectively. It also named *WHEAT LUX ARRHYTHMO* (*LUX*)*/PHYTOCLOCK 1* (*WPCL1*) and might suppress *Ppd-1* transcription by combining in the promoter region of *Ppd-1*. As observed in *Arabidopsis*, the *WPCL1* gene expresses in the daytime and the transcriptional level of the *WPCL1* gene achieve a high level at night [60, 61]. The recessive *ppd-H1* allele in barley can enhance the circadian rhythm gene *CO* homologs and is associated with a decrease in *FT* expression, which is consistent with the late flowering phenotype. The *T. aestivum Heading date 1* (*TaHD1*) gene, also called *CONSTANS* 2 (*CO2*), encodes a transcription factor with a zinc finger motif, a CCT domain, and nuclear localization signals, and is the homolog of *CO* in wheat, and its expression pattern follows a diurnal rhythm under long days. *TaHD1* can compete with VRN2 to integrate the NF-Y transcription factors to form a complex, and plays an opposing role to VRN2 to equilibrate the decreased transcripts of *Vrn3* that are inhibited by VRN2 [44, 62]. The *T. aestivum GIGANTEA* (*TaGI*) gene works upstream of *CO* and encodes a nucleoplasmically-localized protein that contains 1174 amino acid residues, which is initiated by photoperiod and then expressed in the leaves at the seedling stage. It is controlled by the circadian clock under a light/dark cycle and produces a bulky protein complex that binds to the critical region in the *CO* gene promoter [63, 64]. ODDSOC2 is another MADS box transcription factor, that can indirectly restrain flowering, but when plants express *Vrn1* or long time to prolong cold, the expression level of this gene will be down-regulated [38, 65, 66].

When given wheat enough vernalization and photoperiod, differences exist among wheat varieties that are regulated by *Eps* genes. Narrow-sense earliness per se (*Eps*) of wheat is the third effector that influences wheat heading stage equivalently to the autonomous flowering pathway in *Arabidopsis.* Wheat always displays *Eps* when the seeds are subjected to full vernalization and are grown under long days [12, 67]. No major genes have been cloned for this character, and the molecular mechanisms and wheat cultivars of different alleles/allelic combinations have scarcely been reported. *Eps* genes are supposed to be involved in various periods of wheat heading stage and work independently. The *Eps-Am1* gene was identified as the wheat ortholog of circadian clock regulator *EARLY FLOWERING 3 (ELF3)* in *T. monococcum* [12]. Meanwhile, an *Eps* quantitative trait locus (QTL) mapped on the 1DL based on different types of molecular markers also included *TaELF3*, which had functional similarity to the *T. monococcum Eps-Am1* locus. Premature stop codons or deletion of the *TaELF3* gene results in its failure to function and an early flowering phenotype, and thus may be a repressor of flowering [67–70]. The molecular mechanism by which *Eps* regulates heading stage is unknown, and the location of these genes suggests that they are possibly cross-talking with vernalization genes and photoperiod genes, which maybe overspread their influence. Another important factor influencing wheat heading stage is plant hormones. In *Arabidopsis*, GAs, as a class of hormones, can up-regulate the transcription of *SOC1* (*SUPPRESSOR OF OVEREXPRESSION OF CO1*), which encodes a MADS-box gene to accelerate flowering [70]. *Wheat SOC1* (*WSOC1*) in polyploid wheat is homologous with *SOC1* in *Arabidopsis thaliana*, can partially restore the *Arabidopsis SOC1* mutation. Its expression is induced by GA at the seedling stage, but cannot be regulated by vernalization and photoperiod. This gene was expressed in young spikes initiated before the reproductive transition, but was preferentially expressed in leaves and was associated with the expression of *WAP1/Vrn1* in the flowering pathway [71]. The existence of both GA and *Vrn1* precipitate the up-regulation of *WSOC1* and *WFL* (*Wheat FLO/LFY*) to accelerate process of wheat spike development [72]; *WFL* is a gene identified in the floral meristem associated with young spike development that is widely conserved in both monocots and dicots. In conclusion, both *VRN1* and GA are important to wheat shoot apical meristem and spike development under short days by accelerating the expression of *SOC1–1* and *WFL* [73, 74]. The detailed information and function of genes related to wheat heading stage are described in Table 1.

### Part 2. Abundant genetic variation of signal components associated with wheat heading stage

As a heterogeneous hexaploid crop, wheat genome possesses the abundant genetic variation, such as insertions, deletions, point mutations, copy number variation and haplotype diversity etc. Many studies indicated that genetic variants can alter vernalization requirement and photoperiod response to regulate heading stage and flowering times in wheat [75, 76].

### Genetic variation of vernalization-related genes influence wheat heading stage

As a pivotal regulatory element, the allelic diversity of genes regulating the wheat growth life cycle is extensively adapted towards dealing with constant changes and pressure. The variation in *Vrn1* is the most abundant. The existence of one dominant allele of at least one *Vrn1* gene homolog (*Vrn-A1*, *Vrn-B1*, *Vrn-G1*, or *Vrn-D1*) determines the spring growth habit in wheat. Several important domains exist in the promoter region of the *Vrn1* gene, for example, CArG-box is a common binding site for MADS-box proteins located 180 bp upstream of the transcription initiation site, and *Vrn-box* is a sequence (TTAAAAACCCCTCCCC) of 16 bp that determines whether the wheat requires vernalization to promote flowering. Between the CArG box and *Vrn-box* is a G-box (CACGTG), which is a binding site for bZIP transcription factors [16]. In the pre-mRNA of *Vrn1*, a crucial region contributes to the interaction between TaGRP2 and VRN1. Therefore, the allelic variation of *Vrn1* relies on mutations in the promoter region or the intron1 of the A, B, and D genome. An analysis of the mutations in the *Vrn-A1*, *Vrn-B1,* and *Vrn-D1* genes in accessions with a spring growth habit are shown in Table 2. The number of distinct mutations that were reported in the promoter region or intron1 of *Vrn1* can be described as follows. The *Vrn-A1a* gene has three alleles termed *Vrn-A1a.1*, *Vrn-A1a.2,* and *Vrn-A1a.3*. *Vrn-A1a.1,* and *Vrn-A1a.3,* also known as *Vrn-A1a,* which were identified in hexaploid and tetraploid wheat separately. They are associated with a foldback repetitive element insertion and a duplicated region in the promoter, resulting in an intense impact on the vernalization response. *Vrn-A1a.2* displays a 16-bp deletion and four single nucleotide deletions within the MITE insertion compared to *Vrn-A1a.1* [76, 77]. Six types of *Vrn-A1b* allele variants exist that have 20-bp deletions at 157 bp, and *Vrn-A1b.1*-*Vrn-A1b.5* differ from each other only on account of a polymorphism of the A-tract within the *Vrn-box*, and are indicated in turn as AAAAT, AACCC, AAACC, AAAAC, and AAACA. Nevertheless, *Vrn-A1b.6* takes a “C- > G” transversion in the C-rich fragment than *vrn-A1b4* and *Vrn-A1b.1*. Then *Vrn-A1b.2*, *Vrn-A1b.5*, *Vrn-A1b.6* detected for “spring” but *vrn-A1b.3*, *vrnA1b.4* detected for “winter” variants [76, 77]. *Vrn-A1c*, *Vrn-A1d*, *Vrn-A1e*, *Vrn-A1f,* and a series of allele variants have been characterized in polyploid wheat. Compared with the recessive *vrn-A1* allele, *Vrn-A1c*, *Vrn-A1L*, and *Vrn-A1u* have a deletion in intron1, respectively. A variant with a 7.2-kb deletion in intron1 was identified in the tetraploid cultivar ‘Langdon’, and this allele was named *Vrn-A1L*. *Vrn-A1u* is the variant of the 1.4-kb deletion in intron1 identified in *T. urartu* and polyploid species, and *Vrn-A1ins* is another variant with a 0.5-kb insertion near the 5′ side of intron1 in two spring accessions of *T. monococcum* [76, 78]. *Vrn-A1d*, *Vrn-A1e*, *Vrn-A1f*, and *Vrn-A1h* are four alleles that are missing different lengths of fragments in the promoter region. *Vrn-A1d* has a deletion of 32 bp and this fragment contains the complete HDD region and part of the CArG box, generating a diagnostic 147-bp *Msp I* restriction fragment [79]. Another 54-bp deletion in *Vrn-A1e*, covering the CArG box and HDD regions, was discovered in the *Vrn-A1* promoter region of the *T. turgidum* ssp. *dicoccum* accession ST 27 [78]. The *Vrn-A1f* allele has an 8-bp deletion in the 128 to 120 bp region as well as a 50-bp deletion in the 112 to 62 bp region, which is located in the foldback element insertion present in the G genome in tetraploid wheat species [28]. *Vrn-A1h* has a 20-bp deletion near the CArG- box, resulting in the loss of one *MspI* site [78].

**Table 2:** The detailed information of genes association with the wheat heading-flowering regulatory network

Gene		Accession number of NCBI	Mutation region	Mutation pattern	Phenotype	Ployploid wheat	Reference
*vrn-A1*		AY747600	Promoter	A 222-bp foldback element insertion in the promoter region	Later heading	*T. aestivum* cultivar Triple Dirk C	[79]
*Vrn-A1a*	*Vrn-A1a.1*	AY616458	Promoter	A 222-bp foldback element insertion in the promoter region	Early heading	*T. aestivum* cultivar Triple Dirk C	[17]
	*Vrn-A1a.3*				Early heading		
	*Vrn-A1a.2*	KR782255		A 16-bp deletion and 4 single nucleotide deletions within the MITE insertion when compared to *Vrn-A1a.1*	Early heading	*Triticum compactum* cultivar Tiroler Fruhe Binkel	[76]
*Vrn-A1b*	*Vrn-A1b*	AY616461	A 20-bp deletion at − 157 bp in promoter region	A 20-bp deletion at −157 bp	Early heading	*T. aestivum* cultivar Spring Marquis	[17]
	*Vrn-A1b.1*	KM047646		A-tract within the VRN-box: AAAAT	Early heading	*Triticum turgidum* strain PI 264954	[76]
	*Vrn-A1b.2*	KM047641		A-tract within the VRN-box: AACCC	Early heading	*Triticum dicoccoides* strain PI 233288	[76]
	*Vrn-A1b.3*	KM047647		A-tract within the VRN-box: AAACC	Early heading	*Triticum turgidum subsp. dicoccon* strain UA0300214	[76]
	*Vrn-A1b.4*	KM047651		A-tract within the VRN-box: AAAAC	Early heading	*Triticum dicoccoides* strain PI 466941	[76]
	*Vrn-A1b.5*	KM047652		A-tract within the VRN-box: AAACA	Early heading	*Triticum turgidum subsp.* dicoccon strain UA0300212	[76]
	*Vrn-A1b.6*	KT692944		Takes along a “C- > G” transversion in the C-rich fragment than *Vrn-A1b*	Early heading	*Triticum turgidum subsp. durum* strain CItr 10,024	[76]
*Vrn-A1c*		AY747599	Intron1	Intron 1 deletion in the A genome copy	Early heading	*T. aestivum* cultivar IL369	[79]
*Vrn-A1d*		AY616462	Promoter	A 32-bp deletion, which included the complete HDD region and part of the CArG box.	Early heading	*Triticum turgidum subsp. dicoccoides*	[17]
*Vrn-A1e*		AY616463	Promoter	A 54-bp deletion, which included the CArG box and HDD regions	Early heading	*Triticum turgidum subsp. dicoccum* ST27	[17]
*Vrn-A1f*		DQ146421	Promoter	An 8-bp deletion in the region between −128 and − 120, and a 50-bp deletion in the region between −112 and − 62	Early heading	*Triticum monococcum* strain PI503874	[29]
*Vrn-A1h*		GQ451745	Promoter	A 20-bp deletion near the CArG box	Early heading	*Triticum monococcum subsp. aegilopoides*	[78]
*Vrn-A1i*		KM016790	Promoter	Nucleotide substitution in promoter region	Weak effect on the vernalization response	*Triticum turgidum* strain PI 208912	[76]
*VRN-A1u*		GQ451737	Intron1	A 1.4-kb deletion in intron1	Later heading	*Triticum urartu* isolate Tu54	[78]
*vrn-B1*		AY747604	Promoter		Later heading	*T. aestivum* cultivar Triple Dirk C	[79]
*Vrn-B1a*		AY747603	Intron1	6850-bp deletion at + 836 bp	Early heading	*T. aestivum* cultivar Triple Dirk B	[79]
*Vrn-B1b*		FJ766015	Intron1	6850-bp deletion at + 836 bp and 37-bp deletion at + 7992 bp	\	T. aestivum	[80]
*Vrn-B1c*		HQ593668	Intron1	The deletion of 0.8 kb coupled with the duplication of 0.4 kb in intron1	Early heading	*T. aestivum*	[81]
*Vrn-B1ins*		HQ130482	Intron1	817 bp deletion from 798 to 1614 bp, and 432 bp (from 798 to 1614 bp upstream) duplicated	Early heading	*T. aestivum* cultivar Saratovskaya29	[82]
		KR782252	Promoter	A retrotransposon insertion in the promoter	Early heading	*Triticum turgidum* cultivar Zerdakia	[76]
*vrn-D1*		AY747606	No.	No.	Later heading	*T. aestivum* cultivar Triple Dirk C	[79]
*Vrn-D1a*		AY747597	Intron1	A 4235-bp deletion in intron1 (625–4859 bp)	Later heading	*T. aestivum* cultivar Triple Dirk E	[79]
*Vrn-D1b*		JQ406528	Intron1	Deletion in intron 1 identical to *Vrn-D1a* allele and a single nucleotide mutation C to A at − 161 bp in CArG box in promoter region	Later heading	*T. aestivum*	[83]
*Vrn-D1c*		KP721800	Promoter	An 174-bp fragment was inserted into the 5′-UTR at − 601 bp (relative to ATG) of the *Vrn-D1* gene	Early maturity	*T. aestivum*	[85]
*Vrn-D1s*		KF800714	Intron1	An 844-bp insertion which is a novel transposable DNA element in intron1		*Triticum spelta* strain PI 348700	[84]
*vrn-B3*		DQ890162	Promoter	A 5295-bp repetitive element insertion that is absent in the CS allele associated with late flowering	Later heading	*T. aestivum* cultivar Chinese Spring	[18]
*Vrn-B3a*		DQ890165	Promoter	A 5295-bp repetitive element inserted 591 bp upstream from the start codon	Later heading	*T. aestivum* cultivar CS(Hope7B)	[18]
*Vrn-B3b*		JN627519	Promoter	An exact 890-bp fragment was inserted into the 5’ UTR (untranslated region) at − 429 bp	Later heading	*T. aestivum*	[93]
*Vrn-B3c*		JQ082311	Promoter	A 20-bp deletions at −3543 bp and a 4-bp deletion at − 3591 bp compared with *Vrn-B3a*	Later heading	*T. aestivum* cultivar Ji874–109	[93]

Note, No. Means there are no more information refer to

In the *Vrn-B1* locus, one distinct deletion of 6850 bp occurs in intron1 in the *Vrn-B1a* mutant, position is counting start with intron1 in TDB (Triple Dirk B, *vrn-A1/Vrn-B1/vrn-D1*). TDB is a spring wheat that carries a dominant *Vrn-B1* allele, and TDC (Triple Dirk C, *vrnA1/vrnB1/vrnD1*) carries recessive alleles at the three *Vrn-1* loci with a winter growth habit. In addition to this large deletion, two single nucleotide polymorphisms (SNPs) in intron1 and intron2 were detected in TDC and TDB (Fu et al., 2005). *Vrn-B1b* is a mutation with a 6850-bp deletion at + 836 bp and a 37-bp deletion at + 7992 bp in TDC [80]. An 817-bp deletion from 798 to 1614 bp, and a 432-bp duplication upstream of this region was found in the *T. aestivum* cultivar Saratovskaya29, suggesting that distinct alterations within the *Vrn-B1c* allele may influence the flanking sequence of intron1 by breaking a putative binding site [81, 82]. Based on the *Vrn-D1* genome, four alleles have been identified and three of them occur in intron1, while only one allele was found in the promoter region. *Vrn-D1a* was first identified in TDE (*vrn-A1/vrn-B1/Vrn-D1*), which is a spring wheat with the dominant *Vrn-D1* allele. It has a 4235-bp deletion (625–4859 bp) in intron1 compared to TDC [79]. *Vrn-D1b* has the same deletion in intron1 in comparison with the *Vrn-D1a* allele, and in addition, a single nucleotide C to A mutation at 161 bp in the CArG box results in 32-day-later heading [83]. *Vrn-D1s* has an 844-bp insertion in intron1 of *T. spelta* strain PI 348700 and this fragment is a novel transposable DNA element (named DTA_Chimera_KF800714) [84]. The fourth allele constitutes the only one insertion mutant in the promoter region of *Vrn-D1c*. Cultivars with *Vrn-D1c* exhibit earlier heading and flowering than others with the recessive allele *vrn-D1* without vernalization, but display extension heading when treated with prolonged low temperature. The *Vrn-D1c* genotype is important to bread wheat cultivars from the Yellow and Huai Valley areas, and was discovered in Yunong 876 with a 174-bp fragment insertion in the 5’-UTR at 601 bp of the *vrn-D1* gene [85].

Copy number variation (CNV), as a type of gene mutation, play the vital role in the regulation of extensive adaptability. The CNV of *Vrn-A1* alleles investigated also influenced gene expression and showed a slight influence on wheat heading stage and flowering time. One, two, and three *Vrn-A1* copies dictate the time under longer period of exposure to cold conditions that a plant requires to accelerate flowering, and plants with an increased CNV require longer cold treatment to reinforce flowering [75]. A SNP in exon4 of *vrn-A1* affected the QTL of winter wheat development genes, and the alleles *vrn-A1a* and *vrn-A1b* resulted in early flowering in the wheat cultivars Jagger and 2174, respectively [86]. The diverse variation in *VRN1* can be seen in Table 2.

*Vrn2* encodes a zinc finger transcription factor and includes two tandem-duplicated CCT domains, ZCCT1 and ZCCT2, with 76% analogy to a putative zinc finger, *CONSTANS* (*CO*), *CONSTANS-LIKE* (*CO-like*), or *TIMING OF CAB EXPRESSION1* (*TOC1*) [17, 29]. In *Arabidopsis*, the CCT domain and yeast HEMEACTIVATOR PROTEIN2 (HAP2) act similarly as key elements for the HAP2/HAP3/HAP5 complex to bind to CCAAT boxes in the promoter region of their target genes to regulate their expression. Variation in the CCT domain impacting on the functions of proteins CO, TOC1, VRN2, and *PPD-H1* has been identified several in plants, but rare alleles in the CCT domain have been detected in bread wheat [87]. Plants with homozygous recessive *vrn-2* alleles have a spring growth habit, but RNA interference of *ZCCT1* in the hexaploid winter wheat cultivar Jagger can also accelerate flowering [88, 89]. If alleles for winter growth habit are present at all VRN1 loci, then allelic variation for *VRN2* would be detected. The ZCCT1 and ZCCT2 proteins from nonfunctional *vrn-2* alleles have mutations at positions 16, 35, or 39 of the CCT domain that are conserved between the CCT and HEME ACTIVATOR PROTEIN2 (HAP2) proteins. Therefore, both *ZCCT-B2* gene mutations are sufficient to make tetraploid wheat exhibit a spring growth habit [89]. Non-vernalized synthetic *vrn2-null* plants flowered 118 days earlier than the winter control, and showed a limited vernalization response. In the mutant, *Vrn-B2* expressed higher than the *Vrn-D2* and displayed an inhibiting action under partial vernalization, but without vernalization, it only carried *Vrn-B2* or *Vrn-D2* in the heterozygous condition. This suggested that different combinations of *Vrn-B2* and *Vrn-D2* may participate in regulating the vernalization network in mild winter regions [89].

The *T. aestivum* cultivar Chinese Spring (Hope7B) allele *Vrn-B3a* has a 5295-bp repetitive element inserted 591 bp upstream in the promoter region and resulted in early flowering, while the *vrn-B3* allele in Chinese Spring makes wheat flower later. *Vrn-B3b* is an exact 890-bp fragment inserted into the 5′ untranslated region (UTR) at 429 bp identified in one Chinese landrace cultivar Chadianhong. Then, a 20-bp deletion at 3543 bp and a 4-bp deletion at 3591 bp when compared with *Vrn-B3a* in Ji874–109 were discovered and designated as the *Vrn-B3c* allele [18]. The condition of the *Vrn3* alleles is illustrated in Table 2.

Recent studies relating to the characterization of the *Vrn-D4* locus, which arose via the duplication of *Vrn-A1*, discovered the existence of a SNP in the conserved TaGRP2 RIP-3 region that influences the binding ability of TaGRP2 with *Vrn1* [30].

### Diverse variants of photoperiod (*Ppd-1*) genes play different roles in wheat heading stage regulation

Large deletions, insertions, SNP, and copy number variations in *Ppd1-A1*, *Ppd1-B1*, and *Ppd1-D1* have been reported to influence wheat sensitivity to photoperiod [57, 90, 91].

A 2089-bp deletion in the promoter region of *Ppd-D1* in the photoperiod-insensitive wheat cultivar ‘Ciano 67’, regarded as the *Ppd-D1a* allele, was detected and co-segregated with wheat early flowering and has four varieties-*Ppd-D1a.1, Ppd-D1a.2, Ppd-D1a.3* [8, 57, 91]. These four alleles with different length deletions in the promoter region appear in Winter-Abukumawase, “GS-100”, “GS-105”, and Kanak, respectively. Other than barley, the semi-dominant *Ppd-D1a* mutation in wheat permits plants to flower earlier under both SD and LD conditions, while in barley there is no phenotypic effect under SD conditions [8, 57]. Three SNPs were detected in the promoter of wheat cultivars of Winter-Abukumawase (*Ppd-A1b.2*) and Chinese Spring (*Ppd-A1b.1*), with two In/Dels of 1-bp more in *Ppd-A1b.2*. In the 5’ UTR, a 308-bp insertion was identified in Winter-Abukumawase, which results in an insensitive phenotype regarded as *Ppd-B1a.1*. *Ppd-B1b.1* and *Ppd-B1a.2* separately represent a SNP mutation in the promoter region of Chihokukomugi (*Ppd-B1b.1*) and Chinese Spring (*Ppd-B1a.2*) [57, 91] (see Additional file 1). Moreover, some cultivars possessed the photoperiod insensitive *Ppd-D1a* allele and other cultivars had the photoperiod sensitive *Ppd-D1b* allele had been also identified in Huanghuai wheat region [92].

Other modalities of mutations have subsequently been reported. ‘Mercia’ flowers earlier under SD conditions than some photoperiod-sensitive varieties due to the existence of a mariner-like transposable element (MLTE) in intron1, which differs distinctly from the 2089-bp deletion in ‘Ciano 67’ [57].

A recent study indicated that some connection exists between the distribution frequency of *Ppd-D1* haplotypes and the geographical environment of the wheat cultivation regions. A total of eight *Ppd-D1* haplotypes were detected. *HaplotypeI* (*Hapl_I*) was characterized by a 5-bp deletion in exon 7, and the addition of a 2-kb upstream deletion distinguished *Hapl_II* from *Hapl_I*. *Hap_III* has an extra TE insertion in intron1 compared with *Hapl_II*, and *Hapl_IV* only possessed the 5-bp deletion in exon7. *Hap_V* carries a 2-kb deletion in the promoter region, a 5-bp deletion in exon7, and a 16-bp insertion in exon8. *Hap_VI* has a 24-bp plus a 15-bp insertion in the 2-kb upstream region [93]. Two new polymorphism combinations in *Ppd-D1* designated as *Hapl-VII* and *Hapl-VIII* were identified in cultivars that originate from the Yellow and Huai Valley of China. *Hapl-VII* is characterized by the absence of a 2089-bp in exon8 and the presence of both TE in intron1 and 5-bp deletion in exon7, while these are absent in *Hapl-VIII* [92]. An additional file shows the haplotypes of *Ppd-1* in wheat in more detail (see Additional file 2).

Some genes also influence flowering time regulation when they exhibit CNV, but no sequence changes. CNV is closely associated with gene expression and there is less chance of CNV occurring in independent alleles of *Ppd1*. However, copy number variations in the *Ppd-B1* locus have been detected with increased gene numbers that results in earlier flowering [75].

It is clear that diverse mutations of these genes associated with wheat heading stage and flowering influence their expressions, resulting in earlier or later flowering under certain circumstances.

## Conclusions

Based on the current research in wheat, four major pathways exist that regulate wheat heading-flowering time. Vernalization and photoperiod pathways integrate environmental signals to determine the transition from the vegetative phase to the reproductive phase, while *Eps* and GA pathways act as internal stimuli independently of the environmental signals. GA and *Eps* perform throughout wheat growth and development, while vernalization occurs during the transition from the vegetative phase to the reproductive phase during winter, and directly influences spikelet initiation. In addition to vernalization, photoperiod plays a leading role to control flowering begins from spikelet initiation and determines floret initiation and spikelet development, as indicated in Fig. 1.

**Fig. 2:**
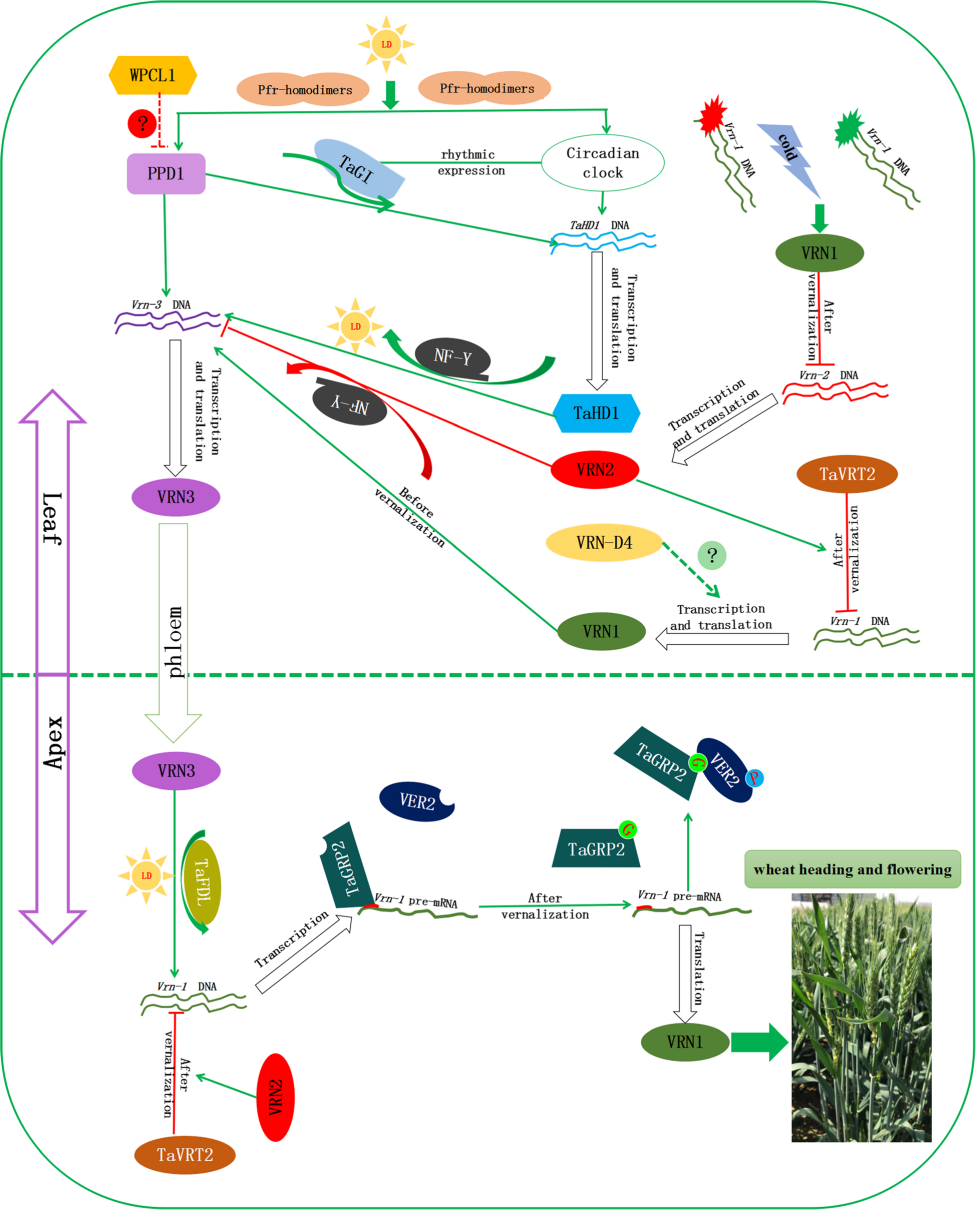
Schematic summary of the wheat heading stage regulatory network. Before vernalization, VRN2 competes with other CCT-domain proteins (like CO2) to interact with NF-Y transcription factors to inhibit the transcription of *VRN3*. Secondly, TaGRP2 can directly bind to this binding site of *VRN1* to prevent transcript accumulation. Thirdly, TaVRT-2 can directly bind to the CArG box of the *TaVRN1* promoter in vivo to inhibit its activity, and this inhibition is enhanced by VRN2. Following vernalization, *VRN1* transcripts were enhanced by changing the ratio of H3K4me3 to active gene transcription. However, the expression level of *VRN2* decreases after vernalization to release *VRN3*, then they can move from the leaves to the apices via the phloem. In the stem apical meristem, the VRN3 protein then forms a functional protein complex with TaFDL to bind the CArG box domain in the promoter of *VRN1* in vitro, leading to transcriptional activation. Meanwhile, phosphorylated VER2 (VER2-P) transfers into the nucleus and then gathers in the shoot tips and young leaves, physically interacting with the RNA-binding protein TaGRP2, which is O-GlcNAc-modified,to decrease the inhibitory action on *VRN1* expression. Furthermore, the expression levels of TaVRT2 and VRN2 decrease, and VRN1 gradually accumulates. Finally, the expression level of VRN1 is significantly enhanced to accelerate flowering. *VRN-D4*, as a duplicated copy of *VRN1*, expresses in the leaves and accumulates after prolonged exposure to low temperature, and can directly or indirectly influence *VRN1* among the three vernalization genes the earliest, but has less effect than *Vrn-A1*. Long-day (LD) induces the accumulation of physiologically active Pfr (PHYB:PHYC heterodimers and PHYC:PHYC homodimers) and then activates the transcription of *PPD1* and circadian clock output genes *CO2/TaDH1,* and the *VRN3* transcript can be promoted by PPD1 and CO2. However, PPD1 is inhibited by WPCL1, which is a flowering negative regulator, but the interaction mechanism of these two genes is unknown. TaGI, which is controlled by the circadian clock under a light/dark cycle, works on the upstream of CO and produces a bulky protein complex with other suspected proteins, binding to the critical region in the *CO* gene promoter to induce its transcription. *Eps* genes work throughout wheat growth and development via an unknown pathway, Green arrows represent promotion, red arrows represent inhibition in the signal pathway

A better understanding of the genetic regulatory mechanism of wheat heading stage assisted breeding. In the case of vernalization, *Vrn1*, *Vrn2,* and *Vrn3* generate a positive feedback loop and were assisted by *TaGRP2*, *VER2*, *TaVRT2,* and *TaFDL2,* constituting a vernalization regulatory network initiating wheat heading stage and flowering. Then, the photoperiodic pathway cross-talks with the vernalization pathway via *Ppd1*, *TaPHYC*, *TaHD1*, *WPCL1* and *TaGI*. *WSOC* and *LHY* also regulate wheat heading stage and flowering time independently via the GA pathway. A schematic summary of the wheat heading stage regulatory network is depicted in Fig. 2. These genes encode members of multitudinous transcription factors, for instance, MADS-box families, some MYB transcription factors, types of Myc and zinc finger families, RNA-binding proteins, and other protein families, and can directly or indirectly interact with each other, thereby supporting the entire wheat heading stage and flowering regulatory network. In spite of lots of researches about heading-flowering based on *Arabidopsis* constructing a relatively prefect network, *Arabidopsis* cannot fully reflect wheat and its relative. Some other components may also exist that participate in the pathway, and the identified genes in the wheat heading-flowering time regulation network are still unclear. Therefore, there are lots of tasks on unknown gene cloning by rapidly growing modern biotechnology, such as high-throughput sequencing, multi-omics profiling, mutant screening and association mapping to offer new exciting insights into future researches. For instance, the VRN2 protein interacts with CO2 (CONSTANTS2) in vitro and can competitively bind with members of the NUCLEAR FACTOR-Y (NF-Y) transcription factor family, but the detailed integration and competitive process of these interactions remains unclear. And if any other hormones participate in the wheat heading stage regulation pathway? The genetic regulation mechanism of hormone and *Eps* is not clear in wheat maybe because of the masking by strong influence of vernalization and photoperoid, *Eps* will be important for fine-tuning some of these traits and understanding their basis is the next major step.

Our knowledge of the regulatory network of wheat heading-flowering can be applied to targeted breeding or other means, including adjusting the duration of the growth phase for the purpose of acclimatizing to different geographical environments.

## Additional files

Additional file 1: **Table S1** Diversity variations of *Ppd-D1* gene in wheat. (DOC 37 kb)

Additional file 2: **Table S2** Haplotype information of *Ppd-D1* gene in wheat. (DOC 26 kb)

## Abbreviations

*AP1*: APETALA1
CNV: Copy number variation
*CO2*: *CONSTANS 2*
*ELF3*: Early Flowering 3
*Eps*: Earliness per se
*FT*: Flowering time
GA: Gibberellic acid
*HAP*: HEME ACTIVATOR PROTEIN
HAP2: Hemeactivator Protein 2
*Hapl_I*: HaplotypeI
LD: Long day
MLTE: Mariner-like transposable element
*NF-Y*: NUCLEAR FACTOR-Y
PHYC: Phytochrome C
*Ppd-1*: Photoperiod
*Ppd-A1*: *Photoperoid-A1*
*Ppd-B1*: *Photoperoid-B1*
*Ppd-D1*: *Photoperoid-D1*
PRR: Pseudo-response regulator
SAM: Shoot apical meristem
SD: Short day
*SOC1*: Suppressor of overexpression of CO1
SVP: SHORT VEGETATIVE PHASE
*TaGI*: *T. aestivum* Gigantea
*TaGRP2*: *T. aestivum* glycine-rich RNA binding protein 2
*TaHD1*: *T. aestivum Heading date 1*
*TaVRT-1*: Wheat vegetative to reproductive transition-1
*TaVRT-2*: Wheat vegetative to reproductive transition-2
*TOC1*: Timing Of Cab Expression1
UTR: Untranslated region
*VER2*: vernalization-related 2
*Vrn1*: Vernalization 1
*Vrn2*: Vernalization 2
*Vrn3*: Vernalization 3
*WAP1*: Wheat APETALA1
*WPCL1*: *Wheat LUX Arrhythmo* (*LUX*)*/Phytoclock 1*
