# Supplementary material for: Gene regulatory network and abundant genetic variation play critical roles in heading stage of polyploidy wheat
Source: BMC Plant Biol. 2019 Jan 3;19:6. doi: 10.1186/s12870-018-1591-z (PMC6318890; doi:10.1186/s12870-018-1591-z)
Supplement: Supplementary file 1 — Table S1 Diversity variations of Ppd-D1 gene in wheat. (DOC 37 kb) [file 12870_2018_1591_MOESM1_ESM.doc]

Table S1：The diverse variants of *Ppd-1* in polyploid wheat

| Gene | NCBI No. | Mutation points | Mutation pattern | Phenotype | Ployploid | Reference |
| --- | --- | --- | --- | --- | --- | --- |
| *PPD-A1a.1* | AB646973.1 | Promoter | A 1085-bp deletion between nucleotides -1420 to -336 in CS | Insensitive | Chihokukomugi | [91] |
| *PPD-A1a.2* | No. | Promoter | A deletion of 1,027 bp share common region in -1,193 and -336 with PPD-A1a.1 | Insensitive | GS-100 | [91] |
| *PPD-A1a.3* | No. | Promoter | A deletion of 1,117 bp share common region in -1,193 and -336 with PPD-A1a.1 | Insensitive | GS-105 | [91] |
| *PPD-A1b.1* | No. | Promoter | 3 SNPs in the 5’ upstream | Sensitive | Chinese Spring | [91] |
| *PPD-A1b.2* | AB646972.1 | Promoter | 3 SNPs in the 5’UTR and 2 InDels of 1bp | Sensitive | Winter-Abukumawase | [91] |
| *PPD-B1a.1* | AB646974.1 | Promoter | *Ppd-B1a.1* of W-AB has a 308-bp insertion between nucleotides -734 and -733 | Insensitive | Winter-Abukumawase | [91] |
| *PPD-B1a.2* | No. | Promoter | A SNP was detected in the 5' UTR | Insensitive | Chinese Spring | [91] |
| *PPD-B1b.1* | AB646975.1 | Promoter | A SNP was detected in the 5' UTR | Sensitive | Chihokukomugi | [91] |
| *PPD-D1a* | DQ885767 | Coding region | A 2,089-bp deletion between nucleotides -2,146 and -58 | Insensitive | Ciano 67 | [57] |
| *PPD-D1a.1* | AB646976.1 | Promoter and exon8 | A 2,089-bp deletion between nucleotides -2,146 and -58 and a 16 bp deletion in exon8 | Insensitive | Winter-Abukumawase, winter wheat cultivars in Huanghuai wheat region | [57, 93] |
| *PPD-D1b* | DQ885770 | Exon7 | A 5-bp deletion in exon7 | Sensitive | Norstar | [57] |
| *PPD-D1b.1* | No. | Promoter and exon8 | A SNP in promoter region and a 16-bp deletion in exon8 | Sensitive | Chinese Spring | [57] |
| *PPD-D1b.2* | AB646977.1 | Promoter, exon7 and exon8 | A 5-bp deletion in exon7 with a SNP in promoter region and a 18-bp deletion in exon8 | Sensitive | Chihokukomugi, winter wheat cultivars in Huanghuai wheat region | [57, 93] |

Note, No. Means there are no more information refer to.
