# Supplementary material for: Gene regulatory network and abundant genetic variation play critical roles in heading stage of polyploidy wheat
Source: BMC Plant Biol. 2019 Jan 3;19:6. doi: 10.1186/s12870-018-1591-z (PMC6318890; doi:10.1186/s12870-018-1591-z)
Supplement: Supplementary file 2 — Table S2 Haplotype information of Ppd-D1 gene in wheat. (DOC 26 kb) [file 12870_2018_1591_MOESM2_ESM.doc]

| Haplotype | Meaning | Description | Growing Condiation | Reference |
| --- | --- | --- | --- | --- |
| *HaplotypeI* | A 5-bp deletion in exon7 | The highest expression level among the six Ppd-D1 haplotypes | Asia, Oceania and Mexico, Huanghuai wheat region | [92-93] |
| *haplotypeII* | A 5-bp deletion in exon7, a 2-kb upstream deletion | Varieties with a heading time intermediate between I and III | Asia, Huanghuai wheat region | [92-93] |
| *HaplotypeIII* | A 5-bp deletion in exon7, a 2-kb upstream deletion, an extra TE insertion in intron1 | Lowest expression level among the six Ppd-D1 haplotypes | Europe and North America, Huanghuai wheat region | [92-93] |
| *haplotypeIV* | The only 5bp deletion in exon7 | Being 5% higher than those of haplotypes II and III, but lower than haplotype I | Across all continents | [92-93] |
| *HaplotypeV* | A 2-kb deletion in the promoter region, a 5-bp deletion in the exon7 and a16-bp insertion in the exon8 | Varieties with a heading time intermediate between I and III | Ancient haplotypes | [92] |
| *HaplotypeVI* | One more 24-bp plus 15-bp insertion in the 2 kb upstream region | / | Ancient haplotypes | [92] |
| *Hapl-VII* | Absence of 2089 bp in exon8 and presence of both TE in intron1 and a 5 bp in exon7 | / | Yellow and Huai Valley of China | [93] |
| *Hapl-VIII* | Absence of 2089bp in exon8, TE in intron1 and 5 bp in exon7 | / | Yellow and Huai Valley of China | [93] |

Table S2：The haplotypes of *Ppd-1* in wheat
